# Sexual Behavior of Married Young Women: A Preliminary Study from North India

**DOI:** 10.4103/0970-0218.39677

**Published:** 2008-07

**Authors:** Ajit Avasthi, Rajinder Kaur, Om Prakash, Anindya Banerjee, Lata Kumar, P Kulhara

**Affiliations:** Department of Psychiatry, Postgraduate Institute of Medical Education and Research (PGIMER), Chandigarh - 160 012, India; 1 Department of Pediatrics, Postgraduate Institute of Medical Education and Research (PGIMER), Chandigarh - 160 012, India

**Keywords:** Female sexual behavior, knowledge and attitude, practices, sexual difficulties

## Abstract

**Background:**

There are significant gaps in the scientific literature concerning female sexual behavior and attitudes surrounding sexuality, which have definitive implications on public health and clinical work.

**Aim:**

To study the sexual behavior of young married Indian women.

**Materials and Methods:**

The study group comprised 100 consecutive women attending the Department of Pediatrics for the care of noncritical children in a multispecialty, tertiary care teaching hospital setting in North India. Current levels of sexual functioning and satisfaction were assessed by using the Brief Index of Sexual Functioning for Women (BISF-W). All participants were also administered a translated and culturally adapted instrument called Sex Knowledge and Attitude Questionnaire-II (SKAQ-II).

**Results:**

Peno-vaginal sex continues to be considered the most desired and actually performed sexual activity for arousal and orgasm, followed by kissing and foreplay. Difficulties while performing sexual activity, in the form of physical problems, were faced by 17% of the participants. The participants displayed adequate sexual knowledge and favorable attitude towards sexuality as measured by SKAQ-II.

**Conclusion:**

The present study is a preliminary effort to understand the contemporary female sexual behavior, knowledge and attitude by employing standard instruments. Still further studies are required in this area.

## Introduction

Human sexuality is the sum total of an individual's biological constitution, life experiences, knowledge, behavior and attitudes; it is influenced by a myriad of physical, psychological, interpersonal, and cultural factors.(1,2)

Human sexuality has numerous symbolic meanings to an individual, and it is important for the clinicians to understand human sexuality. Despite advances in the treatment of human sexual problems, various lacunae remain in our knowledge of human sexuality. In particular, our knowledge of female sexuality has consistently lagged behind that of male sexuality. In fact, relatively little is known about the relationship among sexual behavior, sexual attitudes, sexual fantasies, and marital functioning of women.(3)

In a study performed immediately after the independence of India, sexuality was considered a taboo, and sexual matters were generally not discussed in the family.(4) In many parts of India, a large number of young girls with low literacy levels were married in their early adolescence. Indian girls were found to lack the independent authority for the control of their sexuality or reproduction.(5) Post marriage, the control of female sexuality shifted from the father to the husband. The lack of adequate knowledge in the young women about sexual matters and contraception resulted in early and successive pregnancies and sexual disharmony.(6–8)

In India, since the 1980s, there has been an apparent transition in the attitude of people toward sexuality. Expressions and feelings that would have otherwise been termed as scandalous and in the need of being tamed to adhere to socially accepted rules, values, and practices, are now accepted as natural.(9) More people are accepting the fact that sexuality plays a crucial role not only in procreation but also for pleasure. In surveys, a significant minority of the population have reported homosexual orientation and behavior.(10,11)

It is important to realize that in the last 40 years, considerable scientific studies have been conducted on human sexuality. However, scientific research in the area of sexuality in India is scant and, if studied, they have almost exclusively focused on male sexual disorders.(12,13) Sexual problems of women have not received adequate attention from the researchers. Worldwide, female sexual dysfunction (FSD) is a highly prevalent problem for 38%-63% of women.(14)

A careful review of Indian literature has revealed that except the reports of Agarwal(15)—a study on 17 cases of female frigidity—and those of Kulhara and Avasthi(16) who reported 13 cases of females from a sample size of 464 patients with sexual disorders, this area has remained untouched.

Therefore, keeping in view the abovementioned limitations and the nonavailability of data from India with regard to this important area of human behavior, we decided to study the sexual functioning of women and the relationship between sexual functioning of the women and their knowledge and attitudes regarding sexuality.

## Aim and Objectives

1. To study the sexual behavior of young Indian married women
2. To assess their sexual knowledge and attitude towards sexuality
3. To identify the disorders of sexual functioning in the sample studied

## Materials and Methods

This study was a cross-sectional survey on 100 healthy female participants, consecutively selected after they fulfilled the inclusion criteria and informed consent were obtained; these women were selected from those attending the Department of Pediatrics, in a multispecialty, tertiary care, central government funded teaching hospital in North India for the noncritical care of their children.

### Inclusion criteria

1. Age between 20 and 40 years
2. Married and staying continuously with the spouse at least since the last three months

## Instruments

### 1. Brief Index of Sexual Functioning for Women (BISF-W)(17)

We used a 22-item, self-report instrument for the assessment of current levels of female sexual functioning and satisfaction. This instrument assesses areas such as frequency of sexual behavior, fantasy, masturbation; sexual preferences; partner satisfaction; sexual anxiety; sexual performance difficulties; and sexual satisfaction.

### 2. Sex Knowledge and Attitude Questionnaire (SKAQ-II)(18)

This self-administered questionnaire comprises two parts: knowledge and attitude. The 35-item knowledge-part consists of dichotomous scoring with the maximum attainable score of 35. The attitude part has 20 items, scored on a three-point linear scale (0–2), with maximum obtainable total score of 40. Higher the scores, the better the knowledge and more liberal is the attitude.

#### Procedure

The abovementioned instruments were administered to each participant by a female psychologist (RK), given the sensitivity of the subject area being studied. Self-administration was preferred, but to those who required assistance/clarification, the same was not denied. Confidentiality and anonymity was ensured as a part of the study protocol, and all authors except the interviewer (RK) were blind to the identity of the study participants. To maintain anonymity, identification data was not recorded on the BISF-W and SKAQ-II. Participants manifesting sexual functional difficulties were provided the option of thorough clinical assessment in the Psychosexual and Marital Clinic of the Department of Psychiatry, PGIMER, Chandigarh, for the diagnosis and management of any sexual dysfunction/disorder as per the criteria of the International Statistical Classification and Related Health problems 10^th^ Revision (ICD-10) (World Health Organization, 1992).(19)

## Results

### (a) Socio-demographic characteristics

We studied a total of 100 married women in the age group 20–40 years. A majority of women (90%) were residing in the urban and suburban areas. In terms of years of education, 10.55 years was the mean of the sample. Most of the women were housewives (84%), followed by professionals (9%), clerks (4%) and students (3%). The monthly family income for the majority of the participants (69%) was less than Rs. 6000 (belonging to nonaffluent, low and middle socioeconomic status).

### (b) Female sexual functioning

All participants described their sexual orientation as heterosexual. Most (96%) considered sex as an important aspect of life and were also satisfied with their own body (98%). A majority of the participants were able to communicate their sexual desires or preferences to their spouses. More than half of the sample (58%) admitted having inhibition or anxiety while performing sexual activity. The same percentage of the sample reported that usually spouses took the initiative for sexual activity, while the remaining together initiated the activity. Most of participants (93%) admitted that they performed sexual activity more than or as much as desired by their partners; three-fourths of them accepted sexual advances by their spouse with pleasure. A majority of them (66%) admitted deriving pleasure from sexual experience at more than 75% times, while most of them (95%) revealed that both partners were satisfied with their sexual relationship.

### (c) Sexual practices – [Table 1]

**Table 1:** Percentages of various sexual activities desired/engaged in and their association with sexual arousal/orgasm as measured by the BISF-W

Sexual activity	Desired sexual activity	Sexual activity engaged in	Sexual activity for sexual arousal	Sexual activity for orgasm (more than 75% times)
Peno-vaginal sex	56	95	86	84
Kissing	47	95	40	06
Foreplay	42	76	55	15
Mutual masturbation	29	62	40	11
Oral sex	07	10	09	04
Anal sex	00	01	01	00
Sexual dream/fantasy	00	28	00	01

Peno-vaginal intercourse was considered as the predominant mode of sexual behavior (56%) followed by kissing (47%), foreplay (42%), mutual masturbation (29%) and oral sex (7%). The most common activity leading to sexual arousal was peno-vaginal intercourse, followed by foreplay, mutual masturbation, kissing and oral sex. Orgasm was achieved predominantly during peno-vaginal intercourse (84%). Other sexual behaviors assessed in this study were anal sex and sexual fantasy, which did not appear to significantly impact the sexual desire, arousal or orgasm.

### (d) Disorders of sexual functioning – [Table 2]

**Table 2:** Disorders of sexual functioning

Problem encountered	No. of participants
Difficulties in sex (total)	17
Headache after sexual activity	10
Difficulty reaching orgasm	9
Painful intercourse	7
Lack of vaginal lubrication	5
Vaginal tightness	5
Bleeding after intercourse	3
Vaginal infection	2
Attribution of problems	17
Own health problems	14
Lack of privacy	8
Spouse's health problems	4
Conflict with spouse	4

A total of 17% participants encountered one or more difficulties during sexual activities in the form of headache after sexual activity (10%), difficulty in reaching orgasm (9%), painful intercourse (7%), lack of vaginal lubrication (5%), vaginal tightness (5%), bleeding after intercourse (3%) and vaginal infection (2%). Some participants (14%) attributed these difficulties to their own health problems; further, lack of privacy (8%), spouse's health problems (4%) and conflict with spouse (4%) were the other stated reasons. None considered their sexual difficulty sufficiently significant to demand a thorough clinical examination.

### (e) Sexual attitude and knowledge scores

The participants of the study had adequate sexual knowledge (mean score, 16; SD, 5.58) and liberal attitude (mean score, 31.48; SD, 6.04). A majority of them (62%) were able to communicate their sexual desires to their partners.

## Discussion

This is perhaps the first study in India which has systematically enquired into female sexuality and its relationship with knowledge and attitudes toward sexuality. The majority of the sample has adequate sexual knowledge and a fairly liberal attitude. Moreover, many of the study participants displayed positive dispositions toward greater sexual assertiveness and a wide range of sexual activities. These findings, contrary to the traditional prototype of the Indian woman, may be explained partly in the light of younger age and urban preponderance in the sample; however, the possibility of an evolving social change in female sexuality is pertinent.

None of the respondents admitted any homosexual orientation. Claims of an open society made in previous reports have been noted in men(9) or in couples.(10) Possibly, homosexuality still requires a social sanction in India, and female homosexuality is still not acceptable.

The patterns of sexual behavior reported were not considerably different from those in the Western data, albeit slightly lower rates of masturbation were noted. Given the potential value of kissing, foreplay and mutual masturbation as an alternative to high-risk sexual practices and as potential vehicles to orgasm, investigations of factors governing these methods of physiological sexual fulfillment are required. In our study, oral and anal sex were not popular modes of behavior, which probably reflects the cultural bias against such practices. The rates of oral sex in our sample (10%) resembled that reported by a study from rural China.(20)

Difficulties while performing sex, in the form of physical problems, were faced by 17% of the participants. This is lower than the rates reported in previous studies from the West as well as Asia. There are several reasons for this low rate. In our study, the participants belonged to younger age groups than the comparable groups reported in other studies; Asian studies have shown that female sexual dysfunction (FSD) increases with age.(14) Self-reports about sexual dysfunction, especially in face to face interviews are subject to underreporting bias arising from concerns of social stigmatization.(21) Cultural differences may also play a role since the rates of FSD are lower in Asian women than their Western counterparts. Finally, married women are at a lower risk for sexual dysfunction compared to nonmarried women.(21)

The pattern of physical problems (e.g., headache) and the attribution is more relevant to Indian families and their living conditions. In Western samples, lack of interest, lubrication difficulties and difficulty in reaching orgasm is frequently reported, but headache is probably a socioculturally acceptable mode of presentation, rather akin to somatization as an expression of depressive symptoms. It is noteworthy that none of the affected participants volunteered for tratment of any diagnosable sexual disorder. The reasons may be sampling in non-help-seeking population, fear of labeling and stigmatization, low emphasis on female sexual satisfaction or a poor attitude toward health care professionals. These factors require evaluation in future studies.

The present study is a preliminary effort to understand the contemporary female sexual knowledge, attitude and practices, using standard instruments. The study, however, has some limitations that should be considered while interpreting the findings. The sample used was not community based and the size was too small to be representative of the general population. In this study, no scales for social desirability were used.

Indian society is multicultural and multilingual, with a wide range in the income and standard of living among the population. It is expected that these factors could have impacted the findings. Among these factors, we did not record the caste and religion of the participating participants, and the sample was not sufficiently large to study the impact of these and other relevant sociodemographic factors such as class affluence and the level of education on sexuality and sexual problems. We hope that future research will address these important issues.

Nevertheless, Indian women appear to be rejecting the traditional Indian repressive standards of sexual functioning. Most young Indian women now have liberal attitudes toward varied sexual behaviors, which could be regarded as the components of their usual sexual practice. These results may provide an impetus for further studies on female sexual attitudes and behavior, and their association with sexual dysfunction. Longitudinal studies are necessary to document the changes in society and value systems with regard to this sensitive issue. Increased awareness of current sexual behaviors and attitudes can enable health professionals fulfill the requirements of women with problems in normal sexual functioning.

## References

[1] Lief HI, Reed DM (1976). Sex education in Medicine.

[2] Gabbard GO, Gabbard GO (2001). Mind and brain in psychiatric treatment. Treatments of psychiatric disorders.

[3] Segraves RT (2002). Female sexual disorders: Psychiatric aspects. Can J Psychiatry.

[4] Kapadia KM (1955). Marriage and family in India.

[5] Kumari R (1995). Rural female adolescence: Indian scenario. Soc Change.

[6] Gupta M (1994). Sexuality in the Indian subcontinent. Sex Marital Ther.

[7] Meyer JJ (1930). Sexual life in ancient India: A study in the comparative history of Indian culture.

[8] Russel-Brown P, Rice JC, Hector O (1992). The effect of sex education on teenagers in St. Kitts and Nevis. Bull Pan Am Health Organ.

[9] Nath JK, Nayar VR, RT Francoeur India (Bharat). The international encyclopedia of sexuality.

[10] Savara M, Sridhar CR (1992). Sexual behaviour of urban educated Indian men: Results of a survey. J Fam Welf.

[11] Raizada A, Gupta SB, Kumar A (2002). Sexual practices other than peno-vaginal sex: Perceptions and practices in an urban community. Indian J Community Med.

[12] Avasthi A, Basu D, Kulhara P, Banerjee ST (1994). Psychosexual dysfunction in Indian male patients: Revisited after seven years. Arch Sex Behav.

[13] Verma KK, Khaitan BK, Singh OP (1998). The frequency of sexual dysfunctions in patients attending a sex therapy clinic in North India. Arch Sex Behav.

[14] Hisasue S, Kumamoto Y, Sato Y, Masumori N, Horita H, Kato R (2005). Prevalence of female sexual dysfunction symptoms and its relationship to quality of life: A Japanese female cohort study. Urology.

[15] Agarwal AK (1977). Frigidity: A clinical study. Indian J Psychiatry.

[16] Kulhara P, Avasthi A (1995). Sexual dysfunction on the Indian subcontinent. Int Rev Psychiatry.

[17] Taylor JF, Rosen RC, Leiblum SR (1994). Self-report assessment of female sexual function: Psychometric evaluation of the brief index of sexual functioning for women. Arch Sex Behav.

[18] Avasthi A, Varma VK, Nehra R, Das K (1992). Construction and standardization of a sex knowledge and attitude questionnaire (SKAQ), in simple Hindi, for North Indian population. Indian J Psychiatry.

[19] World Health Organization (1992). The ICD-10 classification of mental and behavioural disorders: Clinical descriptions and diagnostic guidelines.

[20] Liu H, Xie J, Yu W, Song W, Gao Z, Ma Z (1998). A study of sexual behaviour among rural residents of China. J Acquir Immune Defic Syndr Hum Retrovirol.

[21] Laumann EO, Paik A, Rosen RC (1999). Sexual dysfunction in the United States: Prevalence and predictors. JAMA.

